# Whole-Genome Sequences of Two Multidrug-Resistant Acinetobacter baumannii Strains Isolated from Patients with Urinary Tract Infection in Ghana

**DOI:** 10.1128/MRA.00270-19

**Published:** 2019-08-01

**Authors:** Nicholas Agyepong, Usha Govinden, Alex Owusu-Ofori, Mushal Allam, Arshad Ismail, Torunn Pedersen, Arnfinn Sundsfjord, Sabiha Essack

**Affiliations:** a Antimicrobial Research Unit, Discipline of Pharmaceutical Sciences, University of KwaZulu-Natal, Durban, South Africa; b School of Medical Sciences, Kwame Nkrumah University of Science and Technology/Komfo Anokye Teaching Hospital, Kumasi, Ghana; c Sequencing Core Facility, National Health Laboratory Service, National Institute for Communicable Disease, Johannesburg, South Africa; d Norwegian National Advisory Unit on Detection of Antimicrobial Resistance, Department of Microbiology and Infection Control, University Hospital of North Norway, Tromso, Norway; e Department of Medical Biology, Faculty of Health Science, UiT—the Arctic University of Norway, Tromso, Norway; University of Delaware

## Abstract

Multidrug-resistant Acinetobacter baumannii is a major nosocomial pathogen. We describe the whole-genome sequences of two multidrug-resistant Acinetobacter baumannii strains isolated from hospitalized patients in the intensive care unit at Komfo Anokye Teaching Hospital in Ghana. The isolates carry multiple resistance genes, including those for β-lactams, sulfonamides, aminoglycosides, and tetracycline.

## ANNOUNCEMENT

Acinetobacter baumannii is an opportunistic nonfermentative Gram-negative bacterium and has emerged as a multidrug-resistant nosocomial pathogen, posing a treatment challenge to clinicians. Common infections caused by the pathogen include pneumonia, bacteremia, and urinary tract infection (UTI), particularly among immunocompromised patients and patients subjected to invasive procedures such as mechanical ventilation (1, 2). Carbapenem-resistant A. baumannii is listed among the Gram-negative pathogens prioritized by the World Health Organization (WHO) to guide research, discovery, and development of new antibiotics (3). Studies conducted in Ghana implicated the bacterium in 10.1% and 1.6% of all Gram-negative bacterial UTIs (4) and all bacterial infections (5), respectively. In this study, we report on two A. baumannii strains isolated from the urine and a urethral swab from hospitalized patients with UTI in the intensive care unit (ICU) of the Komfo Anokye Teaching Hospital in Ghana.

Urine and urethral swab samples collected from the patients were inoculated directly and streaked on CHROMagar *Acinetobacter* (CHROMagar, France) medium plates and incubated at 37°C for 24 hours. Acinetobacter species appeared as bright-salmon-red colonies on agar plates for identification and isolation. Single colonies were picked and further identified by a Vitek 2 (bioMérieux, France) automated system and confirmed by matrix-assisted laser desorption ionization–time of flight mass spectrometry (MALDI-TOF MS) (Bruker Daltonics Gmbh, Bremen, Germany). Antimicrobial susceptibility of the isolates demonstrated resistance to multiple antibiotic classes/agents, including ciprofloxacin, tetracycline, gentamicin, and trimethoprim-sulfamethoxazole. The isolates were grown on nutrient agar (Oxoid, England) at 37°C for overnight incubation prior to genomic DNA extraction. DNA was extracted from single colonies using a PureLink microbiome DNA purification kit (catalog number A29790; Invitrogen) according to the manufacturer’s standard protocol. Libraries were prepared from 1 ng DNA using a Nextera XT DNA library preparation kit (catalog number FC-131-2001; Illumina) according to the manufacturer’s instructions. Paired-end sequencing with a dual index (8 bp) and 2 × 150-bp cycles was performed using an Illumina NextSeq 550 system. Sequences were quality trimmed and sequencing adapters were removed with Trimmomatic version 0.35 in paired-end mode (6). For A. baumannii P26-70 and P26-77, the total number of sequence reads was 3,275,974 and 3,525,171, respectively. After assembly using SPAdes version 3.9.1 (7), quality scores were assessed using QUAST version 4.6.0 software (8). The assembled genomes were classified taxonomically by multilocus sequencing typing (MLST) using PubMLST version 2.7 software (9), and acquired antimicrobial resistance genes were identified using the ResFinder Web server version 3.1 (10). The total assembly length, coverage depth, *N*_50_ value, GC content, and sequence type for the two A. baumanii strains, 151 and 198, are shown in Table 1. Analysis of antimicrobial resistance genes by ResFinder version 3.1 (10) revealed the presence of multiple genes encoding for resistance to β-lactams, sulfonamides, aminoglycosides, and tetracycline in both A. baumanii strains (Table 1). For all software, default parameters were used.

**TABLE 1:** Whole-genome sequencing and bioinformatics analyses

Isolate no. (strain)	MLST	Coverage (×)	Total length (bp)	No. of contigs	*N*_50_ (bp)	G+C content (%)	Antibiotic resistance genes:			
							Aminoglycosides	β-Lactams	Sulfonamides	Tetracycline
P26-70 (151)	ST1418	172	4,031,913	99	126,198	38.95	*aadB-like*	*blaADC-25-like, blaCARB-8-like, blaOXA-91*	*sul2*	*tet(39)*
P26-77 (198)	ST1418	213	4,024,532	86	140,192	38.94	*aadB-like*	*blaADC-25-like, blaCARB-8-like, blaOXA-91*	*sul2*	*tet(39)*

The genomes were annotated using the National Center for Biotechnology Information (NCBI) Prokaryotic Genome Annotation Pipeline (PGAP) software revision 4. 2 (11). Strain 151 revealed a total of 3,999 genes, including 3,839 protein-coding genes, 66 RNAs, one copy each of 5S, 16S, and 23S rRNA, 59 tRNAs, and 4 noncoding RNA (ncRNA) genes. A total of 3,970 genes, with 3,828 protein-coding genes, 61 RNAs, one copy each of 5S, 16S, and 23S rRNA, 54 tRNAs, and 4 noncoding RNA (ncRNA) genes, were detected for strain 198. The resistance genes and sequence type shared by both isolates suggest the dissemination of the clone in the ICU, possibly attributable to suboptimal adherence to infection control. The genome sequences of clinical multidrug-resistant A. baumannii isolates from an ICU in a Ghanaian teaching hospital may serve as reference strains for future genome sequences from Ghana and Africa as a whole given the paucity of whole-genome sequence data on the continent.

### Data availability.

The complete genome project (BioProject number PRJNA411997) has been deposited at GenBank under the accession numbers NXHM00000000 and QTKC00000000 for strains 151 and 198, respectively. The raw reads have been submitted to the NCBI Sequence Read Archive (SRA) under the accession numbers SRX5417000 and SRX5417001 for 151 and 198, respectively. These are the first versions of these genome sequences.
